# Are GMI gangliosidosis and Morquio type B two different disorders or part of one phenotypic spectrum?

**DOI:** 10.1002/jmd2.12204

**Published:** 2021-03-18

**Authors:** Sandra D. K. Kingma, Berten Ceulemans, Sandra Kenis, An I. Jonckheere

**Affiliations:** ^1^ Centre for Metabolic Diseases University Hospital Antwerp, University of Antwerp Edegem, Antwerp Belgium; ^2^ Department of Pediatric Neurology Antwerp University Hospital, University of Antwerp Edegem, Antwerp Belgium

**Keywords:** genotype‐phenotype, GMI gangliosidosis, intermediate phenotype, lysosomal storage disorder, Morquio type B, pathophysiology

## Abstract

Monosialotetrahexosylganglioside (GMI) gangliosidosis and Morquio type B (MorB) are two lysosomal storage disorders (LSDs) caused by the same enzyme deficiency, β‐galactosidase (βgal). GMI gangliosidosis, associated with GMI ganglioside accumulation, is a neurodegenerative condition characterized by psychomotor regression, visceromegaly, cherry red spot, and facial and skeletal abnormalities. MorB is characterized by prominent and severe skeletal deformities due to keratan sulfate (KS) accumulation. There are only a few reports on intermediate phenotypes between GMI gangliosidosis and MorB. The presentation of two new patients with this rare intermediate phenotype motivated us to review the literature, to study differences and similarities between GMI gangliosidosis and MorB, and to speculate about the possible mechanisms that may contribute to the differences in clinical presentation. In conclusion, we hypothesize that GMI gangliosidosis and MorB are part of one phenotypic spectrum of the same disease and that the classification of LSDs might need to be revised.

## 1 INTRODUCTION

GMI gangliosidosis and Morquio type B (MorB) are lysosomal storage disorders (LSDs), caused by β‐galactosidase (βgal [E.C.3.2.1.23]) deficiency. GMI gangliosidosis, currently classified as a Sphingolipidose, is caused by the accumulation of monosialotetrahexosylganglioside (GMI) ganglioside in several organs, in particular the brain.^1^ It is a neurodegenerative disorder that is classified in three groups with decreasing severity: type I (infantile, MIM230500), II (juvenile, MIM230600), and III (adult form, MIM230650). It is characterized by psychomotor and locomotor regression, seizures, visceromegaly, cherry red spot, strabismus, and facial and skeletal abnormalities.^2^, ^3^ It is diagnosed by an abnormal pattern of oligosaccharides in urine. MorB (Morquio type B, MIM253010), or mucopolysaccharidosis (MPS) type IVB, is caused by accumulation of the glycosaminoglycan (GAG) keratan sulfate (KS), which is most commonly present in bone, cartilage, and cornea. It is characterized by devastating skeletal deformities, including massive spondylo‐epiphyseal dysplasia, short stature, sternal protrusion, odontoid hypoplasia, and severe genua valga. Furthermore, it presents with corneal clouding and cardiac valve disease. The central nervous system (CNS) is typically not affected.^1^, ^2^

Although GMI gangliosidosis and MorB present with a different phenotype and have been described as different clinical entities, there are some reports of patients with intermediate phenotypes between GMI gangliosidosis and MorB.^4^, ^5^, ^6^, ^7^, ^8^, ^9^, ^10^, ^11^, ^12^, ^13^ Also, there is substantial overlap in clinical, biochemical, and genetic characteristics between the disorders.

Earlier reports have described genotype‐phenotype correlation and factors that contribute to the clinical heterogeneity. A comprehensive review on the molecular basis of GMI gangliosidosis and MorB by Callahan^1^ in 1999 offered some hypotheses for the difference between the two diseases, which are also discussed later in the present paper. Also, a very important study in this field was provided by Caciotti et al.^2^ Using in silico analysis and three‐dimensional analysis of *GLB1* (using a model derived from the structure of *Penicillum* and *Bacteroides* because the tertiary structure of *GLB1* was not discovered yet), genotype‐phenotype correlation in a considerable amount of GMI gangliosidosis patients and some MorB patients was studied. They also commented briefly on intermediate phenotypes. However, a report on all previous published cases of intermediate phenotypes in addition to the description of two new patients, and a comprehensive review focused on mechanisms underlying intermediate phenotypes has not been published before.

## 2 CASE HISTORIES

### 2.1 Patient 1

**FIGURE 1:**
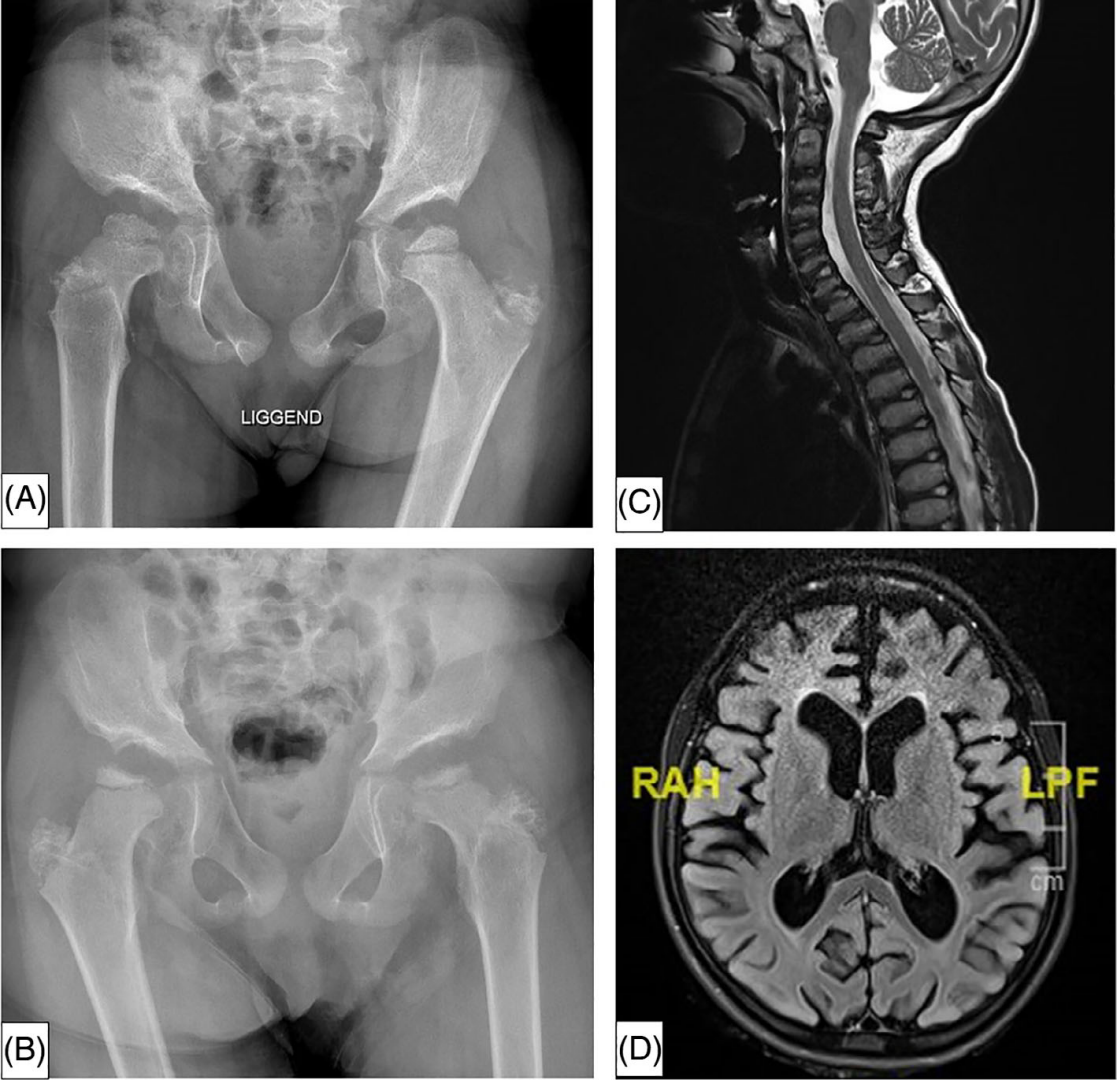
Patient 1 showed ilium hypoplasia, delay of femoral epiphyseal nuclei ossification and coxa valga on X‐ray, A, at the age of 7 years. Epiphyseal dysplasia and flattened femoral heads were apparent on X‐ray at the age of 10 years old, B, MRI studies showed dysplastic cervical vertebral bodies and platyspondyly, C, at the age of 9 years, and enlargement of the ventricular system and extensive cortical atrophy at 13 years old, D

The patient was the first child of unrelated Maroccan parents. She presented at our hospital at the age of 7 years because of psychomotor delay and frequent falls. Her development had been normal until the age of 1.5 years. After that, some delay in psychomotor development and language skills became apparent. Also, she had surgery for strabismus at the age of 3. On physical examination, a short trunk, webbed neck, and kyphosis were observed. She had dysostosis multiplex on X‐ray, as well as hypoplasia of the ilium, delay of femoral epiphyseal nuclei ossification and coxa valga (Figure 1A). Magnetic resonance imaging (MRI) studies showed dysplastic cervical vertebral bodies and platyspondyly (Figure 1C). Further investigations showed an abnormal pattern of oligosaccharides in urine, suggesting GMI gangliosidosis. A mildly elevated urinary excretion of KS, 15.6 mg/mmol (normal limits 0.7‐5.5 mg/mmol) was also observed, indicative for Morquio type A or B. βgal activity was below the detection limit. This confirms the diagnosis of βgal deficiency: either MorB or GMI gangliosidosis, two disorders that are caused by the same enzyme deficiency. Mutation analysis (exons and adjacent introns of *GLB1*), showed a homozygous p.Arg109Trp mutation of the *GLB1* gene, a mutation that has not been described as pathogenic before. Both parents were heterozygous carriers of this mutation. As there is currently no effective therapy available for β‐gal deficiency, symptomatic therapy was started. Today, the patient is deteriorating. She has developed severe epilepsy, epiphyseal dysplasia, hypoplastic ilium (Figure 1B) and MRI shows extensive cortical atrophy (Figure 1D).

### 2.2 Patient 2

Recently, the 8 year younger brother of patient 1 presented with mental regression. He has not been diagnosed previously, because parents refused examination of the siblings of patient 1. The 6‐year‐old boy exhibited dysmorphic features, intellectual disability, delayed motor skills and furthermore, short stature, webbed neck, sternal protrusion and kyphoscoliosis. X‐rays showed dysostosis multiplex, anterior wedging of the lower thoracic and higher lumbar vertebral bodies, hip dysplasia and bullet shaped phalanges. His urinary oligosaccharide pattern was abnormal and βgal activity was below the detection limit. He exhibited the same homozygous p.Arg109Trp mutation as his sister, without the presence of other mutations. The clinical characteristics, oligosacchariduria, βgal deficiency, and *GLB1* gene mutation led to the diagnosis of an intermediate phenotype between GMI gangliosidosis and MorB. Symptomatic therapy was initiated.

## 3 DISCUSSION

The clinical characteristics of our patients obscure the lines between GMI gangliosidosis and MorB, two diseases that are both caused by βgal deficiency. The severe neurological deficits and epilepsy are typical for GMI gangliosidosis, but the extensive bone disease and keratan sulphaturia (KSuria) are typical for MorB. Our patients motivated us to study differences and similarities between these two disorders and to explore possible mechanisms that may cause the intermediate phenotype.

## 4 CLINICAL OVERLAP

In Table 1, we summarized the clinical manifestations of our cases and previously described cases of intermediate βgal deficiency.^4^, ^5^, ^6^, ^7^, ^8^, ^9^, ^10^, ^11^, ^12^, ^13^ All patients with an intermediate phenotype exhibited neurological and skeletal manifestations to some extent. Somatic symptoms were less common, or not described.

**TABLE 1:** Clinical phenotype of previously published patients with intermediate βgal deficiency

References	Mutation	Age of onset	Age of diagnosis	Neurological abnormalities	Bone abnormalities	Other	Urine KS/Oligo Enzyme activity
5, 11	p.Tyr333Cys/p.Tyr333Cys (+sibling)	1.5 y	11 y	Severe mental regression, deterioration speech, walk, intellect (1.5 y), few words, unable to walk, incontinence, cervical myelopathy (11 y)	Short stature and femoral necks, sternal protrusion, gibbus, kyphosis, poor development odontoid process/atlas, flat C3‐5, platyspondyly, wedging Th‐L vertebrae, short clavicles, wide thoracic cavity, long/narrow limbs, narrow pelvis (11 y)	Corneal clouding	+/+ 2.4%
5, 11	p.Tyr333Cys/p.Tyr333Cys (+sibling)		8 y	Severe mental regression, deterioration speech, walk, intellect (first years), few words, unable to walk, incontinence, cervical myelopathy (8 y)	Short stature, flat C3‐4, wedging middle/lower Th and L vertebrae, kyphosis, short clavicles, irregular epiphyses, dysplastic femoral heads, thin limbs		+/+ 2.7%
8	p.Tyr83His/p.Arg482Cys	7 y	15 y	Low school achievements, bilateral reduced vision	Scoliosis (7 y), shorter left leg (8 y), growth retardation (11 y), short neck, barrel‐shaped chest, kyphoscoliosis		+/+ 3–10%
6	p.Thr82Met/p.Tyr270Asp	2 y	4 y	Loss ability to speak, tetraspastic (10 y)	Dysostosis multiplex (4 y)		0.23 nmol/mg/min
6	p.Arg201His/p.His281Tyr	1.25 y	5 y	Slight developmental delay, gait abnormalities (3 y)	Impairment skeletal development, dysostosis multiplex (6 y)		0.38 nmol/mg/min
12	p.Gly438Glu/p.Gly438Glu		8 y	Dull, IQ borderline normal	Short stature, scoliosis, limited ROM joints	Corneal clouding	+/ 0%
13	p.Gly438Glu/p.Gly438Glu (+sibling)	7 y	19 y	Loss of motor skills (7 y), dystonia, dysarthria, swallowing difficulty, drooling, choreoathetoid movements, myoclonic jerks, moderate intellectual impairment	Platyspondyly, kyphoscoliosis, femoral dysplasia (17 y)		2.7%
13	p.Gly438Glu/p.Gly438Glu (+sibling)	3 y	15 y	Impaired gait/speech (3 y), dystonia, dysarthria, swallowing difficulty, drooling, choreoathetoid movements, myoclonic jerks, moderate intellectual impairment (13 y), unable to walk (14 y)	Flattening and wedging vertebrae, odontoid hypoplasia, and dysplasia of the hips and shoulders (13 y)	Aortic regurgitation (13y)	8.7%
10	p.Arg201His/p.Ser149Phe	4 y	14 y	Mild intellectual deficit	Dorsolumbar kyphoscoliosis, facial abnormalities, odontoid dysplasia, genua valga		−/− 3.6%
4	p.Arg201His/p.Gly311Arg	2.5 y	5 y	Gait abnormalities (2.5 y), mild learning difficulties, processing delay (5 y)	Prominent thoracolumbar junction (2.5 y), wedging L1/2 (5 y)	Corneal clouding	+/+ 0%
9	p.Thr384Ser/ p.Thr500Ala	3 y		Learning difficulty (7 y), dysarthria/dystonia (17 y), dysphagia (20 y), extrapyramidal signs	Skeletal deformities (3 y), short stature, hip dysplasia, epiphyseal dysplasia, platyspondyly, wedging L1, kyphoscoliosis		3.8%
9	p.Arg59His/ p.Thr500Ala	1 y		Development delay (1 y), dysarthria/dystonia (22 y), extrapyramidal signs, pyramidal signs	Short stature, hip dysplasia, epiphyseal dysplasia, platyspondyly, wedging L1, scoliosis		7.7%
9	p.Arg59His/ p.Arg201His	7 y		Neurological regression, dysarthria (7 y), extrapyramidal signs, pyramidal signs	Short stature, hip dysplasia (12 y), epiphyseal dysplasia, platyspondyly, lumbar spondylolisthesis (14 y), wedging L1, scoliosis	Angio‐keratoma	4.2%
9	p.Arg59His/ p.Arg201His	7 y		Neurological regression, dysarthria (7 y), extrapyramidal signs	Skeletal deformities (9 y), short stature, hip dysplasia, epiphyseal dysplasia, platyspondyly, wedging L1, scoliosis		5.1%
9	p.Thr500Ala/ c.1722‐1727AinsG	1 y		Dysarthria (13 y), dysphagia (21 y), extrapyramidal/ pyramidal signs	Skeletal deformities (14 mo), short stature, hip dysplasia, epiphyseal dysplasia, platyspondyly, wedging L1, kyphosis		10%
^9^	p.Gly311Arg/p.Thr500Ala	3.5 y		Development delay (3.5 y), dysarthria (4 y), dystonia (11 y), dysphagia (18 y), extrapyramidal/ pyramidal signs	Short stature, hip dysplasia, epiphyseal dysplasia, platyspondyly, wedging L1, scoliosis, kyphosis		11.5%
^9^	p.Thr500Ala/ c.1722‐1727AinsG			Dysarthria, dysphagia (16 y), pyramidal signs	Skeletal deformities (12 y), short stature, hip dysplasia, epiphyseal dysplasia, platyspondyly, wedging L1, scoliosis		9.1%
This report	p.Arg109Trp/p.Arg109Trp (+sibling)	1.5 y	7 y	Developmental delay (1.5 y), extensive mental regression, strabismus (3 y), epilepsy (12 y), extensive cortical atrophy	Short trunk, webbed neck, kyphosis, dysostosis multiplex, dysplastic vertebrae and vertebral disks, epiphyseal dysplasia, ilium hypoplasia, delay of femoral epiphyseal nuclei ossification, coxa valga, platylospondyly	Strabismus	+/+ 0%
This report	p.Arg109Trp/p.Arg109Trp (+sibling)	4 y	5 y	Intellectual deficit, delayed motor skills, dysarthria	Short stature, webbed neck, sternal protrusion, dysostosis multiplex, hip dysplasia, kyphoscoliosis, vertebral wedging, bullet shaped phalanges	Dysmorphic features	0%

Abbreviations: C; cervical, Th; thoracal, L; lumbar, KS; keratan sulfate, Oligo; Oligosaccharide pattern, ROM; range of motion, y; years old, mo; months old.

### 4.1 Neurological manifestations

Neurological manifestations of type I (infantile) GMI gangliosidosis include hypotonia followed by hypertonia, seizures, neurological regression, spastic quadriparesis and early death. The more attenuated phenotypes are characterized by less pronounced neurological disease (type II; juvenile) to extrapyramidal signs such as dysarthria and dystonia (type III, adult).^3^ In contrast, the MorB phenotype is characterized by skeletal disease but patients typically lack neurological disease.^12^, ^14^ In Table 1, neurological symptoms of patients with an intermediate phenotype between GMI gangliosidosis and MorB are summarized. A significant amount of patients were primarily diagnosed with MorB, but developed neurological symptoms, ranging from delayed achievements of milestones to intellectual disability, dystonia and spasticity. These patients were classified as having intermediate phenotypes or “MorB plus disease”.^7^ It seems, therefore, that intermediate patients exhibit symptoms similar to the spectrum of neurological manifestations of GMI gangliosidosis.

### 4.2 Skeletal manifestations

MorB is characterized by a pronounced skeletal phenotype, including midface hypoplasia, mandibular protrusion, short stature with disproportionally short trunk, kyphoscoliosis, sternal protrusion, coxa and genua valga, platyspondyly, vertebral wedging, odontoid hypoplasia, narrow spinal canal, hip dysplasia and dysplasia of metaphysis, and epiphysis of long bones.^7^ The skeletal manifestations in GMI gangliosidosis have a significant overlap with MorB. Skeletal manifestations of GMI gangliosidosis may include short stature, hip and epiphyseal dysplasia, platyspondyly, anterior wedging of vertebral bodies, kyphoscoliosis and the typical constellation of radiographic features, generally referred to as dysostosis multiplex.^1^, ^9^

Patients with intermediate phenotypes were reported as having slightly less severe skeletal abnormalities in comparison with classical MorB patients,^6^ but more severe as compared to GMI gangliosidosis patients (Table 1). Such observations are, however, difficult to objectify. For instance, patients with infantile GMI gangliosidosis usually die before the age of 3 years. If patients would live longer, their skeletal manifestations might have developed into worse than observed in MorB patients.^1^ Thus, it is very difficult to distinguish GMI gangliosidosis and MorB patients based on skeletal manifestations alone.

### 4.3 Somatic manifestations

Classically, patients with GMI gangliosidosis show a generalized disease phenotype with in addition to neurological and skeletal disease: coarse facial features, dysmorphic features, cherry‐red spot, strabismus, visual deficits, hepatomegaly, and splenomegaly.^2^, ^3^ The MorB phenotype is characterized by primarily skeletal disease but may include some somatic manifestations such as joint laxity, cardiac valve disease, and corneal clouding.^12^, ^14^ Patients with an intermediate phenotype between GMI gangliosidosis and MorB did not frequently have other somatic symptoms, or these were not described in the reports. Three cases of corneal clouding, one case of angiokeratoma and one case of cardiac valve disease were reported.

## 5 OVERLAP IN STORAGE MATERIAL

GMI gangliosidosis is characterized by accumulation of GMI gangliosides and is diagnosed by an abnormal urinary excretion of oligosaccharides. MorB is characterized by increased urinary KS excretion.^1^ Overlap in storage material has been demonstrated frequently. KS storage has been noted in the liver of a patient with infantile GMI gangliosidosis, but the authors considered the impact to be minimal in comparison with ganglioside accumulation.^1^, ^15^ In addition, there are patients described with a clinical diagnosis of GMI gangliosidosis that had KSuria and KS storage in fibroblasts.^16^, ^17^ Also, GMI gangliosidosis patients with neither KSuria nor oligosacchariduria have been reported.^16^ In MorB, some patients have oligosacchariduria,^7^, ^18^ and some patients lack KSuria.^7^ GMI gangliosides are probably the cause for neurodegeneration in GMI gangliosidosis but are also involved in the pathogenesis of neurological diseases such as Alzheimer's disease.^19^, ^20^ Although there appears to evidence of neuropathology in MorB brain, neither GMI ganglioside nor KS appear to be stored.^21^ There have, however, been few analysis of MorB brain. Patients with an intermediate phenotype between GMI gangliosidosis and MorB had different oligosaccharide and KS excretion patterns (Table 1). If reported, however, most patients had both KSuria and oligosacchariduria.

## 6 PATHOPHYSIOLOGICAL MECHANISMS

Our patients motivated us to review the literature on suggested mechanisms that may contribute to the difficult distinction between GMI gangliosidosis and MorB in our patients and the earlier described patients in literature.

Intermediate phenotypes were reported in patients with the following 13 missense mutations: p.Arg59His,^9^ p.Thr82Met,^6^ p.Tyr83His,^8^ p.Ser149Phe,^10^ p.Arg201His,^4^, ^6^, ^9^, ^10^ p.Tyr270Asp,^6^ p.His281Tyr,^6^ p.Gly311Arg,^4^, ^9^ p.Tyr333Cys,^11^ p.Thr384Ser,^9^ p.Gly438Glu,^12^, ^13^ p.Arg482Cys,^8^ p.Thr500Ala,^9^ and one insertion c.1722‐1727AinsG.^9^ No clear genotype‐phenotype correlation exists, as most of these mutations have been associated with different phenotypes, as summarized in Table 2. p.Arg59His, p.Thr82Met, p.Ser149Phe, p.Tyr270Asp, p.His281Tyr, p.Tyr333Cys, and p.Gly438Glu have been associated with GMI gangliosidosis. p.Tyr83His, p.Arg482Cys, and p.Thr500Ala have been associated with MorB. Finally, p.Arg201His has been associated with both GMI gangliosidosis and MorB. The mutations p.Gly311Arg, p.Thr384Ser, and c.1722‐1727AinsG have, to our knowledge, only been observed once. The patients had an intermediate βgal deficiency phenotype (Table 1).

**TABLE 2:** Mutations associated with intermediate βgal deficiency and their proposed pathophysiological mechanisms

Amino acid	Amino acid change (references)	Nucleotide change	No of patients	Associated phenotype	Location protein	Proposed mechanisms
Mutations associated with intermediate βgal deficiency						
Arg‐59	p.Arg59His^9^	c.176G > A	3 cHZ	Type I ^1^, ^22^	Intradomain protein core,^23^ TIM^23^	Changed shape of ligand binding pocket, affecting ligand recognition^23^
Arg‐109	p.Arg109Trp^a^	c.325C > T	2 HZ		Protein surface,^2^ TIM^23^	Less stable, aggregation prone protein^2^
Arg‐201	p.Arg201His^4^, ^6^, ^9^, ^10^	c.602G > A	5 cHZ	Type II/III,^2^, ^10^, ^24^ MorB^25^	Protein surface,^23^ TIM^23^	Protein folding,^26^ subcellular trafficking and premature degradation^7^, ^10^
Tyr‐333	p.Tyr333Cys^11^	c.998A > G	2 HZ	Type I/II^10^	Ligand binding pocket,^23^ TIM^23^	Direct effect ligand recognition, catalytic activity^7^, ^23^
Gly‐438	p.Gly438Glu^12^, ^13^	c.1313G > A	3 HZ	Type II/III^2^, ^10^	Protein surface^23^	Enzyme complex formation, lysosomal transport,^12^ protein folding, precursor processing^27^
Thr‐500	p.Thr500Ala^9^	c.1498A > G	5 cHZ	MorB^6^, ^10^, ^14^, ^22^	Protein surface^23^	Catalytic activity,^10^ subcellular trafficking^12^
Mutations not clearly associated with intermediate βgal deficiency (one or two times compound heterozygote or in combination with other known associated mutations)						
Thr‐82	p.Thr82Met^6^	c.245C > T	1 cHZ	Type III^24^, ^28^, ^29^	Intradomain protein core,^23^ TIM^23^	Protein folding,^7^ unstable enzyme precursor, premature degradation^27^
Tyr‐83	p.Tyr83His^8^	c.247 T > C	1 cHZ	MorB^22^, ^30^, ^31^	Ligand‐binding pocket,^23^ TIM^23^	Direct effect ligand recognition, catalytic activity^23^
Ser‐149	p.Ser149Phe^10^	c.446C > T	1 cHZ	Type I^32^	TIM^23^	Unknown,^7^ dependent on other mutation^32^
Tyr‐270	p.Tyr270Asp^6^	c.808 T > G	1 cHZ	Type I/III^10^	Ligand‐binding pocket,^23^ TIM^23^	Direct effect ligand recognition, catalytic activity,^7^, ^23^ subcellular trafficking^10^
His‐281	p.His281Tyr^6^	c.841C > T	1 cHZ	Type I/III^2^, ^23^	Protein surface,^23^ TIM^23^	Protein folding,^26^ catalytic activity^7^
Gly‐311	p.Gly311Arg^4^, ^9^	c.931G > A	1 cHZ		TIM^23^	Enzyme substrate interaction^4^
Thr‐384	p.Thr384Ser^9^	c.1150A > T	1 cHZ			Unknown
Arg‐482	p.Arg482Cys^8^	c.1444C > T	1 cHZ	MorB	Interdomain protein core^23^	Interdomain interaction, enzyme complex formation,^23^ complete absence^7^
		c.1722‐1727AinsG^9^	2 cHZ			Unknown

Abbreviations: cHZ, compound heterozygote; HZ, homozygote; TIM; TIM barrel domain.

^a^ This report.

Factors that may influence phenotypic severity and contribute to the occurrence of intermediate phenotypes include mutations in specific sites of the *GLB1* gene, the type of mutation, and mutations that cause alterations in substrate specificity, subcellular trafficking of the enzyme or enzyme complex formation. We discuss these different mechanisms and if possible, we link the suggested pathophysiological mechanisms to mutations that have been associated with intermediate phenotypes. In addition, we state whether these mutation has previously been associated with GMI gangliosidosis or MorB (Table 2).

### 6.1 Influence of mutation site

The mutation site in the *GLB1* gene determines the effect on βgal protein structure and stability. The tertiary structure and thus the identification of essential sites of human *GLB1* have been resolved in 2012 by Ohto et al.^23^. Due to this study, it is possibe to locate the protein defect and provide insight in the effect of individual mutations. The authors mapped disease causing mutations into the three‐dimensional structure of *GLB1* and compared the predicted effect to the known disease phenotype. Patients with type I GMI gangliosidosis (severe phenotype) more often had mutations in the protein core, and in particular in the TIM barrel domain. The TIM barrel domain is responsible for the catalytic activity of the enzyme, and mutations probably lead to severely decreased enzyme activity of βgal. Milder phenotypes were caused by mutations located in the surface regions that probably caused little structural change but may affect enzyme complex aggregation and enzyme stability, leading to decreased but not absent enzyme activity. Mutations that caused MorB were somewhat localized toward the ligand binding pocket and β‐domain 2, but further conclusions were not made. However, the effect of some mutations could not be explained. For instance, mutations associated with the most severe phenotypes were sometimes located on the surface of the protein.^23^

Ohto et al.^23^ also mapped some mutations that are associated with intermediate phenotypes into the three‐dimensional structure of *GLB1*. These mutations are not limited to a certain site, but are localized all over the protein: first, in the ligand‐binding pocket, second, in the protein core and finally, on the protein surface. In the next section, we summarize the structural changes caused by the mutations that are associated with intermediate phenotypes, as described in the paper of Ohto et al.^23^

First, the mutations p.Tyr83His, p.Tyr270Asp, and p.Tyr333Cys are localized in the ligand binding pocket. The side‐chain of Tyr‐83, the changed amino acid in p.Tyr83His,^8^ is almost buried in the bottom of the ligand‐binding pocket. The OH group of the side chain forms hydrogen bonds with several other amino acids, and furthermore, its side chain is stacked over the side chains of several other amino acids.^23^ The amino acid Tyr‐270, which is changed into Asp in the mutation p.Tyr270Asp,^6^ normally forms hydrogen bonds with another amino‐acid, Glu‐268, fixing the amino acid sequence into its appropriate position in the ligand‐binding pocket to catalyse reactions.^23^ Tyr‐333, the changed amino acid in mutation p.Tyr333Cys,^11^ forms a part of the lateral side of the ligand‐binding pocket and its OH group interacts with galactose on the ligand.^23^ These mutations therefore may affect the shape of the ligand binding pocket, most likely reducing affinity with the ligand and reducing catalytic activity. In addition, the authors have shown that these mutations are localized in a part of the ligand‐binding pocket that directly contributes to ligand recognition.^23^ A direct effect on ligand recognition would probably have a severe effect on enzyme function. However, these mutations have been associated with different phenotypes: p.Tyr83His with MorB,^22^, ^30^, ^31^ p.Tyr270Asp with type I GMI gangliosidosis,^10^ and p.Tyr333Cys with type I/II GMI gangliosidosis.^10^ Therefore, it is plausible that other factors contribute to phenotypic severity in addition to the effect on ligand interaction and the structural change in the enzyme.

Second, mutations associated with intermediate βgal deficiency that were located in the protein core are: p.Arg59His, p.Thr82Met and p.Arg482Cys. Arg‐59, the amino acid in the mutation p.Arg59His, was almost totally buried inside the protein core. This amino acid forms several interactions that stabilise the structure of the ligand binding pocket. A mutation such as p.Arg59His disrupts these interactions and changes the shape of the ligand‐binding pocket, probably having a severe effect on enzyme function.^23^ p.Arg59His is indeed mostly responsible for a type I GMI gangliosidosis phenotype.^22^ Thr‐82, the amino acid that is mutated in p.Thr82Met, forms intradomain interactions, and forms a hydrogen bond with Ile‐55. The authors did not discuss further effects of the mutation,^23^ but if a hydrogen bond is not formed, it might alter the structure of the protein (core) or protein folding. p.Thr82Met is most frequently observed in patients with a type III GMI gangliosidosis phenotype with some residual βgal activity.^24^, ^28^, ^29^ The amino acid Arg‐482 that is changed in p.Arg482Cys is totally buried in the protein core, forms interdomain interactions, and has several ion and hydrogen bonds. Theoretically, if the residue is changed, organization between domains may be disrupted, decreasing the stability and possibly the ability to form enzyme complexes, which is important for transportation of the enzyme to the lysosome.^23^ p.Arg482Cys has been observed in MorB.^29^, ^30^, ^31^

Finally, the mutations p.Arg201His, p.His281Tyr, p.Gly438Glu, and p.Thr500Ala are located at the protein surface. Arg‐201, which is mutated in p.Arg201His, is located at the protein surface at the lateral side of the TIM barrel domain, far from the ligand‐binding pocket. p.Arg201His does not result in any structural rearrangement, except for the loss of a salt bridge to Asp‐198, an amino acid which mutation is also a known cause of βgal deficiency.^23^ Thus, the p.Arg201His mutation may cause a βgal deficiency phenotype due to the mutation at the TIM barrel domain itself, or by influencing Asp‐198. p.Arg201His is frequently observed in type II GMI gangliosidosis,^2^, ^10^, ^24^ but has also been described in type III GMI gangliosidosis^25^ and MorB.^25^ p.His281Tyr, p.Gly438Glu, and p.Thr500Ala are also located at the surface of the protein, with probably minimal effects on protein structure, but these mutations are not further discussed by Ohto et al.^23^ p.His281Tyr has been associated with type I GMI gangliosidosis,^2^ but also type III.^23^ p.Gly438Glu has been associated with type II and III GMI gangliosidosis.^2^, ^10^ The p.Thr500Ala mutation has been frequently associated with MorB.^6^, ^10^, ^14^, ^22^

In the future, it would be very informative to map the other mutations that are associated with intermediate βgal deficiency, and also the mutation that was found in our patients: p.Arg109Trp.

Differences between phenotypes may also be caused by mutations in parts of the *GLB1* gene that are responsible for the transcription of a splicing variant of βgal. Upon expression of the *GLB1* gene, there are two different transcripts: first, the enzymatically active βgal and second, an alternatively spliced variant of βgal (Sgal or elastin binding protein, EBP). Sgal shares most of its amino acid sequence with βgal but differs from βgal due to the absence of exons 3, 4, and 6 and the presence of 32 other amino acids in exon 5 due to a different reading frame.^1^, ^14^, ^29^ Sgal is catalytically inactive. It does not localisze to lysosomes but is routed to the cell surface where it acts as a chaperone for tropoelastin in all elastin‐producing cells. It protects tropoelastin from aggregation and degradation, thereby facilitating the assembly of tropoelastin upon growing elastic fibres in the extracellular matrix (ECM).^14^, ^33^

Most mutations in the *GLB1* gene will affect both spliced products, βgal and Sgal. Those mutations may result in impaired ECM formation, and a more pronounced bone phenotype.^14^, ^29^ Thus a mixed phenotype of GMI gangliosidosis and MorB might occur. Impaired elastogenesis has been demonstrated in both patients with GMI gangliosidosis type I and MorB.^14^ One study on Sgal described a reduction of elastin deposition in the ECM of fibroblasts of a patient that had a mutation in compound heterozygosity with p.Arg201His,^16^ a mutation that has been observed several times in patients with intermediate βgal deficiency (Tables 1 and 2) and type II GMI gangliosidosis, but also in patients with type I GMI gangliosidosis and MorB.^2^, ^10^, ^24^, ^25^

A relationship between Sgal and disease phenotype has not been proven. Mutations in any βgal phenotype are not limited to the genomic area in the *GLB1* gene that codes for Sgal. Possibly, Sgal contributes to the direction of phenotypic severity, in addition to other factors. The few studies that are reported on Sgal are immunohistochemistry studies on fibroblasts. There have been no studies performed yet on ECM morphology in bone of GMI gangliosidosis or MorB patients. Also, future expression studies of mutant Sgal are needed to determine whether its involvement contributes to the clinical spectrum of βgal deficiency.

### 6.2 Influence of type of mutation

Another factor that may contribute to the differences in phenotype between GMI gangliosidosis, MorB and the intermediate type, may be the type of mutation.

Nonsense mutations and frameshift mutations will result more frequently in loss or change of amino acids and in premature chain termination. This will likely cause loss of enzyme activity and a severe phenotype. To our knowledge, all nonsense mutations and frameshift mutations result in GMI gangliosidosis and not MorB or intermediate phenotypes. However, the vast majority of disease causing mutations in βgal deficiency are missense mutations. And of the known mutations that are associated with an intermediate phenotype, all except one (c.1722‐1727AinsG, insertion) mutations are also missense mutations.

Less severe missense mutations and mutations in areas that cause a shorter but functional protein, result in less stable or misfolded proteins, leading to decreased βgal activity. For instance, p.Arg201His has been shown to not significantly alter enzyme activity, but rather enzyme stability, by studying enzyme activity assays.^10^ Chaperone sensitivity studies may be performed to discover if missense mutations cause misfolded enzymes. p.Arg201His and p.His281Tyr are missense mutations that are associated with intermediate phenotypes, in which enzyme activity was enhanced by treatment with chaperones.^26^ p.Arg201His is frequently associated with type II GMI gangliosidosis^2^, ^10^, ^24^ but also with type III and MorB.^25^ p.His281Tyr is associated with both type I and type III GMI gangliosidosis.^2^, ^23^

Finally, p.Arg109Trp, the missense mutation in our patient, has been suggested to result in a less stable, more aggregation prone protein. This effect, however, has only been hypothesized because of the predicted surface‐exposed site and because the mutation was present together with two other pathogenic mutations in an earlier reported patient with βgal deficiency.^2^

The influence of polymorphisms, deep intronic mutations and modifier genes have also been hypothesized to be able to alter the phenotype. Several polymorphisms in the *GLB1* gene have been described, some of which are known to reduce enzyme activity. Some *GLB1* mutations that are associated with different βgal deficiency phenotypes are observed to coincide with a polymorphism, thereby providing an explanation for the heterogeneity in phenotype in those patients.^1^, ^2^, ^22^, ^34^ In addition, deep intronic variants have been shown in other LSDs (Morquio type A) to be able to impact splicing, and thereby may influence the clinical phenotype.^35^ Modifier genes, thus mutations in another gene not directly related to the disease may unexpectedly influence the phenotype of the disease. Actually, *GLB1* has been shown to be a modifier gene in a mouse model of Krabbe disease, another LSD.^36^ Polymorphisms, modifier genes, and deep intronic variants may also contribute to the occurrence of intermediate phenotypes.

### 6.3 Mutations that influence substrate specificity

Mutations that result in altered substrate specificity might also contribute to the differences between GMI gangliosidosis and MorB. Certain βgal mutations would lead to altered hydrolysis of either GMI ganglioside or KS, thereby determining the phenotype.

A clear example of altered substrate specificity is the p.Trp273Leu mutation. This mutation is the most frequent gene defect in MorB patients and has not been associated with GMI gangliosidosis or intermediate βgal deficiency. It has been confirmed that this mutation affects the degradation of KS more severely than the degradation of GMI ganglioside,^10^ probably because the original amino acid sequence in this mutation, Trp‐273, is important for the binding to either the terminal galactose or disaccharide of specifically KS in the ligand‐binding pocket.^7^, ^23^

Okumiya et al.^30^ investigated substrate specificity of βgal from fibroblasts of both GMI gangliosidosis and MorB patients, by studying the degradation of analogs for GMI ganglioside and KS. The study used a MorB cell line with a p.Tyr83His mutation (associated with intermediate βgal deficiency in addition to MorB) in compound heterozygosity, and a cell line with the p.Trp273Leu mutation (only associated with MorB).^30^ They showed that βgal from both MorB cell lines had a lower hydrolytic activity toward an analog for KS, as compared to an analog for GMI ganglioside. Thus theoretically, mostly KS would accumulate, resulting in a MorB phenotype with only bone abnormalities. In fibroblasts from patients with GMI gangliosidosis, the activity was similar for both analogs. Because the residual enzyme activity for all substrates is low, GMI gangliosides and KS would both accumulate, resulting in a complete spectrum of disease characteristics with severe neurological and bone abnormalities.

Another factor that may influence substrate specificity is the activator protein saposin B. Degradation of GMI gangliosides requires saposin B, which binds the lipid part of GMI ganglioside, facilitates its solubility, and enhances enzyme interaction. KS degradation does not require saposin B binding. βgal mutations at the saposin‐binding site would therefore reduce the ability of the enzyme to degrade GMI ganglioside and would lead to a GMI gangliosidosis phenotype, rather than a MorB phenotype.^1^, ^33^

To date, very limited studies have been performed on the influence of substrate specificity on GMI ganglioside/KS storage and βgal deficiency phenotype. Further information may be essential in understanding the occurrence of intermediate phenotypes.

### 6.4 Mutations that alter subcellular trafficking

After synthesis, βgal travels to the lysosome to exert its function. Alterations in subcellular trafficking may affect enzyme function. βgal cDNA encodes 677 amino acid residues, including a 23 amino acid N‐terminal signaling peptide, which is cleaved upon entry in the endoplasmic reticulum. The βgal precursor is transported to the lysosomal/endosomal compartment and is processed into its mature form, where it forms an enzyme complex with other enzymes.^33^ Subcellular trafficking of the enzyme after synthesis has been studied using immunostaining. Several mutations have been shown to alter subcellular trafficking, including mutations that are associated with intermediate βgal deficiency. βgal in fibroblasts of patients with p.Arg201His, p.Tyr270Asp, p.Gly438Glu, and p.Thr500Ala has been shown to accumulate in the perinuclear area and did not traffic to the lysosomal/endosomal compartment. The protein was most likely degraded in the endoplasmic reticulum.^10^, ^12^ Altered subcellular trafficking may influence phenotypic severity. There is, however, no clear association of altered subcellular trafficking alone with a phenotype yet, as p.Arg201His is associated with type II/III GMI gangliosidosis and MorB,^2^, ^10^, ^24^, ^25^ p.Tyr270Asp with type I/III GMI gangliosidosis,^10^ p.Gly438Glu with type II/III GMI gangliosidosis,^2^, ^10^ and p.Thr500Ala with MorB.^6^, ^10^, ^14^, ^22^

### 6.5 Mutations that alter enzyme complex formation

Alterations in enzyme complex formation due to mutations in the enzyme complex binding site can influence phenotypic severity and may also contribute to intermediate phenotypes.

Synthetized βgal forms an enzyme complex with neuraminidase 1, *N*‐acetylgalactosamine‐6‐sulfate sulfatase (GALNS), and protective protein cathepsin A upon arrival in the endosomal‐lysosomal compartment.^2^, ^33^

The enzymes in the enzyme complex are, if deficient, all responsible for causing a LSD. GALNS is responsible for KS degradation and if deficient, causes Morquio type A. Neuraminidase 1 is, as βgal, responsible for one of the first steps in glycosphingolipid degradation. If deficient, it causes sialic acid accumulation and sialidosis. Finally, cathepsin A protects the other enzymes from degradation. Deficiency causes both βgal and neuraminidase deficiency, which results in the clinical picture of galactosialidosis.^33^

The enzyme complex is essential for enzyme stability and post‐translational processing of the βgal precursor to the mature form and therefore essential for the degradation of GMI ganglioside and KS.^2^, ^12^ Mutations in βgal that influence the enzyme complex may alter the clinical phenotype. The p.Gly438Glu mutation, which is associated with intermediate βgal deficiency and type II/III GMI gangliosidosis, has been shown to cause abnormal complex formation. The presence of relatively high residual activity (6.1%) in the presence of defective enzyme complex formation suggests that the latter mechanism causes the clinical manifestations of βgal deficiency, rather than the low but perhaps still sufficient enzyme activity.^12^

All components in the enzyme complex may influence complex formation and thereby the function of βgal. Therefore, in the presence of a mutation in βgal, an additional modification in enzyme complex binding site of one of the components of the enzyme complex may alter phenotypic severity, even without decreased activity of the individual enzymes.

Previous authors suggested that altered enzyme complex formation has major effects on particularly KS catabolism because most of the enzymes are involved in KS catabolism.^12^, ^33^ There is, however, no evidence yet for an association between KS accumulation in MorB patients and mutations that cause enzyme complex impairment. Furthermore, neither sialidosis, galactosialidosis nor βgal deficiency have been associated with GALNS deficiency, the only enzyme that is exclusively responsible for KS degradation.

Some of the mutations (p.Ser149Phe, p.Thr384Ser, c.1722‐1727AinsG) associated with an intermediate phenotype have not been described more than once, and pathophysiological mechanisms are yet unknown. These mutations coexist in combination with mutations that are more frequently associated with intermediate phenotypes: p.Arg201His and p.Thr500Ala, which were both five times present in compound heterozygosity (Table 1). Therefore, the clinical presentation of these mutations probably depend largely on the other carried mutation. This is also possible for the other mutations that occurred only one or two times in compound heterozygosity, for example, p.Thr82Met, p.Tyr83His, p.Tyr270Asp, p.His281Tyr, p.Gly311Arg.

**FIGURE 2:**
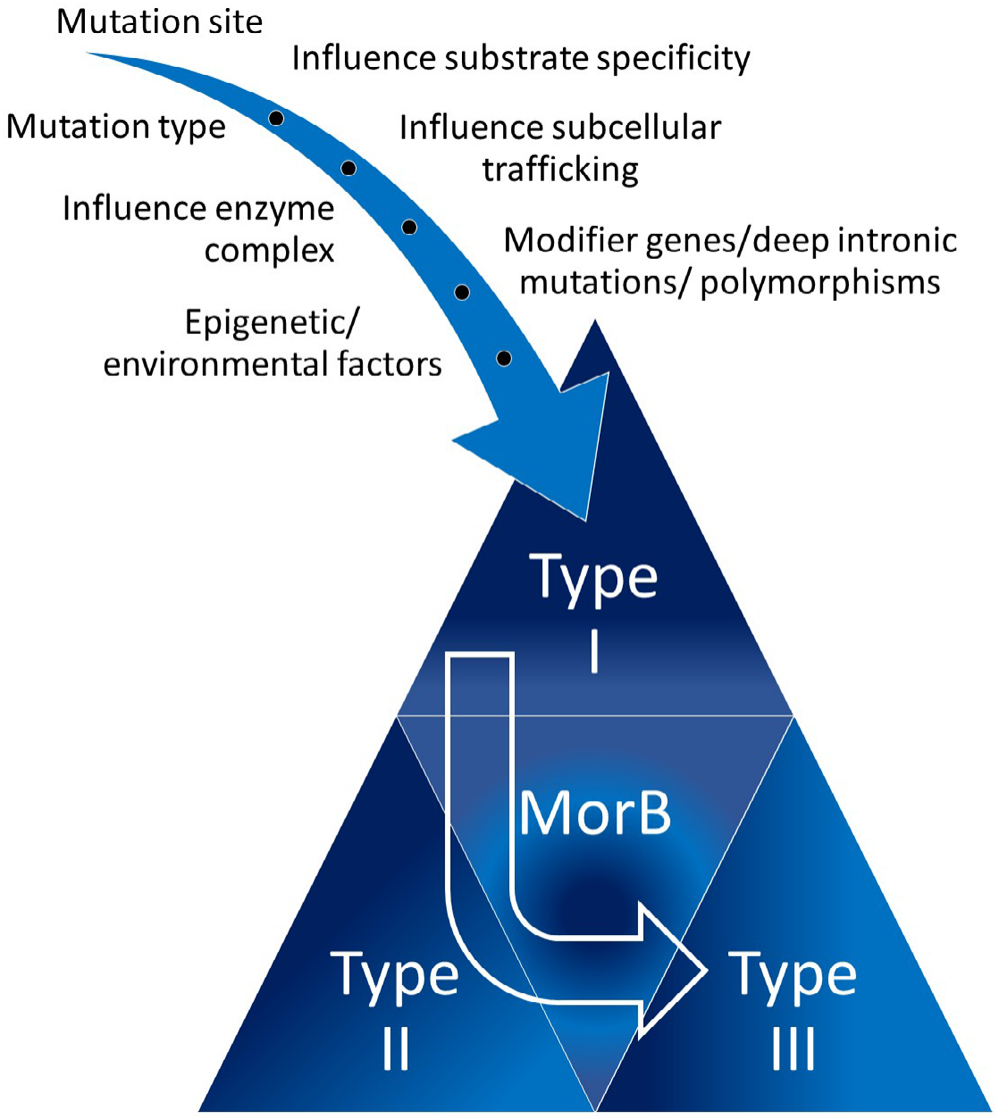
Contributing factors to phenotypic severity and the intermediate phenotype between GMI gangliosidosis and MorB

Most probably, the occurrence of an intermediate phenotype between GMI gangliosidosis and MorB is not caused by only one of the above described mechanisms. It is more likely a concurrence of the influence of mutation site, mutation type, substrate specificity, enzyme trafficking, enzyme complex function, modifier genes, deep intronic mutations, polymorphisms or epigenetic, and environmental factors. This is summarized in Figure 2.

## 7 MUTATION IN OUR PATIENT

A different but interesting discussion is the significance of the p.Arg109Trp mutation observed in our patients. It has been described earlier by Caciotti et al.^2^ but not as a pathogenic mutation. The mutation was predicted to result in a surface exposed residue change. This might make the protein more aggregation prone, which would have a small effect on enzyme activity. In their patient, the mutation was present in combination with two known pathogenic mutations. Also, in silico analysis with sequence alignment between species showed that the mutation site was not highly conserved. Due to these reasons, the mutation was predicted to have a polymorphic nature.^2^ In addition, in gnomAD, the mutation is described in 75 control subjects in a homozygous state with an allele frequency of 0.01.

Thus far, we found no evidence of other mutations that could explain the constellation of clinical manifestations typical for GMI gangliosidosis and MorB, nor the low enzyme activity. The clinical phenotype and the p.Arg109Trp mutation in the two siblings also argues against another (de novo) disease causing mutation. Due to the evidence described above, however, we cannot assume yet that the mutation p.Arg109Trp causes the clinical phenotype, and other factors have to be considered such as deep intronic mutations. cDNA analysis may provide a reason for the conflicting evidence. In addition, expression studies on the p.Arg109Trp mutation would be very interesting.

## 8 CHALLENGES AND FUTURE STUDIES

There have been several studies on clinical, biochemical, and genetic factors that might contribute to the broad clinical spectrum of disease severity in βgal deficiency and the existence of intermediate phenotypes.^1^, ^5^, ^7^, ^16^, ^29^, ^30^ Despite several proposed mechanisms as described in this article, a clear relationship has not been proven. More studies are needed to elucidate the pathophysiological mechanisms behind the difficult distinction of GMI gangliosidosis and MorB. However, several diagnostic challenges have to be met.

First, studying storage of GMI gangliosides and KS in the affected tissues (brain and bone/cartilage, respectively) may be interesting, but these tissues are hardly available. Second, with current available enzyme activity assays, enzyme activities are often below the detection limit, making reliable comparisons impossible. Enzyme activities may be more reliably measured with assays that are optimized for extremely low enzyme activity, such as has been performed for MPS type I.^37^

Third, due to the rareness of the diseases, in particular the intermediate and MorB phenotypes, information on the clinical spectrum, laboratory specimens and biochemical information are lacking. An international patient registry has recently been initiated, in which clinical data but also biological samples are collected.^7^ Studying genotype‐phenotype correlation globally in a large group of patients with βgal deficiency may prove to be essential for better understanding of intermediate phenotypes.

Fourthly, not for all mutations associated with intermediate βgal deficiency, the influence on enzyme architecture has been studied. This may provide more information on the etiology of intermediate βgal deficiency.

Lastly, more research on the influence of the p.Arg109Trp mutation is warranted, for instance gene expression studies.

### 8.1 Classification

Although GMI gangliosidosis and MorB typically have distinct storage products and clinical features, there is in many cases an evident overlap in phenotype, genotype, and storage products. Usually, extremes in phenotypes that share the same enzyme deficiency are classified in the same group, such as in Hurler disease and Scheie disease which are nowadays known as the extremes of a phenotypic spectrum in MPS type I. Careful conclusions about genotype‐phenotype correlations and future research are warranted. However, the overlap between the two diseases suggest in our opinion and some authors before us,^2^, ^38^ that GMI gangliosidosis and MorB, which are both caused by βgal deficiency, might be part of a phenotypic spectrum of the same disease. Earlier, names such as “βgal deficiency” or “MorB plus disease” have been suggested.^7^, ^38^ We propose, however, that the current LSD classification, with MorB as a mucopolysaccharidosis and/or GMI gangliosidosis as a Sphingolipidose, might need to be revised.

## 9 CONCLUSION

GMI gangliosidosis and MorB are currently classified as different LSDs. Both are caused by βgal deficiency and intermediate phenotypes have been described. We reviewed the literature on differences and similarities between these two disorders. Due to the occurrence of intermediate phenotypes, and the overlap between the two disorders, we hypothesize that GMI gangliosidosis and MorB may be part of the phenotypic spectrum of the same disease. We suggest that the classification of LSDs might need to be revised.

## CONFLICT OF INTEREST

Sandra Kingma, Berten Ceulemans, Sandra Kenis, and An Jonckheere declare that they have no conflicts of interest related to this article.

## AUTHORS CONTRIBUTIONS

Sandra D. K. Kingma: designing, conducting, reporting, revising the work described in the article. Berten Ceulemans: reporting, revising the work described in the article. Kenis Sandra: reporting, revising the work described in the article. An I. Jonckheere: designing, reporting, revising the work described in the article.

## INFORMED CONSENT

Informed consent was obtained from all patients for being included in the study.
